# Neonatal hyperglycemia as an independent risk factor for retinopathy of prematurity: a multicenter retrospective cohort study with dose-response analysis

**DOI:** 10.3389/fped.2026.1975596

**Published:** 2026-10-23

**Authors:** Yongming Wang, Xiaoqin Wang, Huijuan Yin, Fengzhi Xu, Wang Xu, Jianping He

**Affiliations:** 1 Neonatology Department, Yinchuan Maternal and Child Health Hospital Affiliated to Ningxia Medical University, Yinchuan, Ningxia Hui Autonomous Region, China; 2 Neonatology Department, People’s Hospital of Ningxia Hui Autonomous Region, Yinchuan, Ningxia Hui Autonomous Region, China; 3 Neonatology Department, General Hospital of Ningxia Medical University, Yinchuan, Ningxia Hui Autonomous Region, China; 4 Neonatology Department, Guyuan Municipal Maternal and Child Health Hospital, Guyuan, Ningxia Hui Autonomous Region, China

**Keywords:** cox proportional hazards model, hyperglycemia, infant, preterm infants, retinopathy of prematurity

## Abstract

**Background:**

Retinopathy of prematurity (ROP) is a major cause of childhood visual impairment. Although neonatal hyperglycemia has drawn research interest, its independent effect on ROP remains controversial. This study explores the association between neonatal hyperglycemia and ROP among infants with gestational age <32 weeks.

**Methods:**

In this multi-center retrospective cohort of 308 preterm infants, multivariable Cox proportional-hazards models adjusted for perinatal factors, maternal comorbidities and neonatal complications. Sensitivity analyses further accounted for invasive/non-invasive ventilation duration, total oxygen therapy time, peak inspired oxygen fraction and glucose infusion rate; Firth penalized regression corrected sparse-data bias. Additional sensitivity analyses examined the dose-response association with neonatal peak glucose levels.

**Results:**

Our findings revealed that hyperglycemic infants had significantly lower birth weights and gestational ages compared to their non-hyperglycemic counterparts. After controlling for confounding variables, hyperglycemia was identified as an independent risk factor for ROP, with an adjusted hazard ratio (aHR) of 2.54 (95% CI: 1.45–4.44, *P* = 0.001). This association persisted after further adjustment for respiratory support variables and glucose infusion rate (aHR=2.46, 95% CI: 1.40–4.31) and in Firth penalized analysis (aHR=2.36, 95% CI: 1.36–4.11). Notably, a pronounced dose-response relationship was detected, with risks escalating markedly at peak glucose levels exceeding 8.33 mmol/L (150 mg/dL) (aHR=2.72) and intensifying further above 10 mmol/L (180 mg/dL) (aHR=3.64), indicating an approximately 50%–80% average risk increase per glucose tier across model specifications (all *P*-trend < 0.001), a gradient that was unchanged after further adjustment for neonatal complications, respiratory support, and glucose infusion rate. In exploratory analysis, severe ROP (stage ≥3, *n* = 13) was not significantly associated with hyperglycemia after penalized adjustment (OR = 1.45, 95% CI: 0.36–7.31).

**Conclusion:**

This study establishes neonatal hyperglycemia as a significant, dose-dependent predictor of ROP and associated multi-organ injury in preterm infants, independent of traditional risk factors. Clinically, this underscores the imperative for stringent glycemic monitoring, with a candidate vigilance threshold of 8.33 mmol/L (150 mg/dL), the clinical benefit of which requires prospective validation. Future prospective studies integrating continuous glucose monitoring and mechanistic investigations are essential to refine glycemic thresholds and optimize metabolic management strategies aimed at enhancing visual outcomes in this susceptible population.

## 1 Introduction

Retinopathy of prematurity (ROP) is a devastating neurovascular disease of the developing retina in preterm infants, characterized by abnormal retinal vascular proliferation (1, 2). It remains a leading cause of preventable childhood blindness worldwide (3). Despite significant advancements in neonatal care, the incidence of ROP is increasing as survival rates of extremely preterm infants improve (4, 5). This trend places a growing burden on affected children, their families, and healthcare systems. Current screening strategies rely primarily on serial fundus examinations, while severe ROP is managed with laser photocoagulation or intravitreal anti-vascular endothelial growth factor (VEGF) agents (6, 7). However, these treatments are invasive, costly, and not without limitations, including potential systemic side effects and the risk of disease reactivation (8, 9). These challenges underscore an urgent need to identify modifiable risk factors for early prevention.

Hyperglycemia, a common metabolic disturbance in preterm infants due to immature insulin secretion and glucose regulation (10). It has emerged as a potential risk factor for ROP. Both experimental and clinical evidence suggests that hyperglycemia may induce retinal vascular damage through mechanisms such as oxidative stress, upregulation of VEGF, and the accumulation of advanced glycation end-products (AGEs) (11, 12). These processes can collectively promote aberrant retinal neovascularization (13, 14). Several observational studies have reported an association between hyperglycemia and ROP, yet the findings remain inconsistent (15, 16). Many of these studies were limited by small sample sizes and inadequate control for critical confounders such as gestational age and birth weight (17, 18). Furthermore, few have rigorously examined the dose-response relationship between hyperglycemia severity and ROP risk (15).

To address these gaps, this study aims to investigate the independent association between neonatal hyperglycemia and the risk of developing ROP. It also explores the potential dose-response relationship between hyperglycemia severity and ROP severity. We hypothesize that hyperglycemia is an independent risk factor for ROP, and that the risk increases with higher blood glucose levels. Elucidating this relationship is crucial. It could enable identification of high-risk infants who may benefit from targeted glucose management and enhanced ROP screening (19, 20).

To test this hypothesis, we conducted a multicenter, retrospective cohort study. This design was chosen to leverage the advantages of a relatively large sample size and the collection of detailed clinical data from multiple centers, thereby enhancing the generalizability of the findings. We employed a rigorous statistical approach, including multivariable Cox proportional hazards regression models to systematically adjust for a comprehensive set of potential confounders, such as gestational age, birth weight, maternal comorbidities, and other neonatal complications. This methodological rigor is essential for isolating the independent effect of hyperglycemia from other known risk factors for ROP.

The primary objective of this study is to determine whether hyperglycemia is an independent risk factor for ROP in a cohort of preterm infants. A secondary objective is to characterize the dose-response relationship between the degree of hyperglycemia and the risk of ROP, providing a quantitative basis for establishing clinical thresholds for glucose control. The findings are expected to provide robust evidence to inform clinical guidelines for blood glucose management in preterm infants. They may also identify a high-risk population for more intensive ROP surveillance, ultimately contributing to the reduction of ROP-related visual impairment.

## 2 Materials and methods

### 2.1 Study population

This study was conducted from January 2024 to June 2025. The study population comprised preterm infants with a gestational age (GA) of < 32 weeks who were born at or transferred to the neonatal units of Yinchuan Maternal and Child Health Hospital, the General Hospital of Ningxia Medical University, Guyuan Maternal and Child Health Hospital, and the People's Hospital of the Ningxia Hui Autonomous Region. The inclusion criteria were as follows: (1) GA < 32 weeks; (2) hospitalization duration ≥ 4 weeks. The exclusion criteria were as follows: (1) major congenital diseases affecting retinal development (e.g., severe brain developmental abnormalities or congenital metabolic disorders) or hereditary ocular diseases; (2) missing critical clinical data or incomplete collection of hyperglycemia exposure information. According to hyperglycemia exposure status, infants were divided into a hyperglycemia-exposed group and a non-exposed group. This study was reviewed and approved by the Ethics Committee of Yinchuan Maternal and Child Health Hospital (Approval No.: 2024–64).

### 2.2 Sample size calculation

Based on previous literature (21), the incidence of retinopathy of prematurity (ROP) was approximately 18% in the non-exposed group and 45% in the hyperglycemia-exposed group. With a two-sided significance level of *α*=0.05 and a statistical power of 1−*β*=0.90. The calculation yielded a minimum sample size of 61 infants per group. Considering a 10%–15% rate of loss to follow-up and incomplete data, at least 70 infants were required per group. The actual sample size enrolled in this study met the above requirements.

### 2.3 Blood glucose measurement and definition of hyperglycemia

Bedside blood glucose screening was uniformly performed at all participating centers using a capillary blood glucose meter (ACCU-CHEK Performa; Roche Diagnostics, Sandhofer Straße 116, 68305 Mannheim, Germany). Blood glucose monitoring was initiated for all enrolled preterm infants upon admission: fasting blood glucose was measured at least once every 8 h (i.e., ≥ 3 measurements per day) for 7 consecutive days. When the glucose infusion rate was < 5 mg/kg/min and the capillary blood glucose measured at any time point exceeded 7 mmol/L (126 mg/dL), venous blood was immediately drawn for confirmatory re-testing. Hyperglycemia was defined as a venous blood glucose concentration > 7 mmol/L (126 mg/dL) confirmed by ≥ 2 independent measurements during hospitalization.

### 2.4 ROP screening and diagnostic criteria

ROP screening was performed in strict accordance with the Chinese Guidelines for Screening of Retinopathy of Prematurity (22). Screening was initiated at a corrected GA of 31 weeks for infants born at < 28 weeks’ GA, and at 4 weeks after birth for infants born at ≥ 28 weeks’ GA. Fundus examinations were performed by ophthalmologists who had undergone standardized training and obtained certification, using the RetCam 3 neonatal digital wide-field fundus imaging system (Clarity Medical Systems, USA), and all images were recorded. ROP staging (stages 1–5) and zones (zones I–III) were determined according to the International Classification of Retinopathy of Prematurity (ICROP), and the presence of plus disease, prethreshold disease, and threshold disease was recorded. All participants underwent regular retinal screening from enrollment until complete retinal vascularization or postmenstrual age of 40 weeks, whichever occurred first. Infants with ROP meeting treatment indications received laser photocoagulation and/or intravitreal injection of anti-vascular endothelial growth factor (anti-VEGF) agents as recommended by the guidelines.

### 2.5 Definitions and diagnostic criteria

The diagnostic criteria for small for gestational age (SGA), periventricular–intraventricular hemorrhage (IVH; diagnosed by cranial ultrasound according to the Papile grading system), bronchopulmonary dysplasia (BPD), hemodynamically significant patent ductus arteriosus (hsPDA), neonatal necrotizing enterocolitis (NEC), and neonatal late-onset sepsis (LOS) were established based on the relevant chapters of Practical Neonatology (5th edition) (23).

### 2.6 Data collection

The following clinical data were retrospectively collected through the electronic medical record system: (1) Maternal and perinatal data: maternal age, pre-delivery body mass index (BMI), mode of delivery (vaginal delivery/cesarean section), antenatal full-course dexamethasone administration (defined as completion of ≥ 4 doses of dexamethasone or ≥ 2 doses of betamethasone), conception via assisted reproductive technology, premature rupture of membranes (PROM), multiple birth (≥2 fetuses), and maternal pregnancy complications (diabetes mellitus [including gestational diabetes mellitus and pregestational diabetes], hypertension [including gestational hypertension and pregestational hypertension], and thyroid disease); (2) neonatal baseline data: sex, birth weight, GA, SGA status, and 5-minute Apgar score; (3) hyperglycemia exposure data: occurrence of hyperglycemia and peak blood glucose level; (4) respiratory support and nutrition data: duration of invasive mechanical ventilation, duration of non-invasive ventilation, total duration of oxygen therapy, peak inspired oxygen fraction (FiO₂), and the mean glucose infusion rate (GIR, mg/kg/min) during the 24-hour period preceding the initiation of glucose-lowering management (for hyperglycemic infants) or during the corresponding postnatal day (for normoglycemic infants); (5) neonatal complication data: IVH (any grade), hsPDA, BPD (any grade), NEC (Bell stage ≥ II), and LOS (defined as a positive blood or cerebrospinal fluid culture more than 72 h after birth accompanied by clinical symptoms).

### 2.7 Outcome measures

The primary outcome was ROP occurrence, defined as the first diagnosis of ROP (any stage/zone) by an ophthalmologist according to the ICROP criteria and the Chinese Guidelines for Screening of Retinopathy of Prematurity (2014) during the period from birth to the end of follow-up. Outcome determination was based on RetCam 3 fundus imaging findings, and the date of the first diagnosis was taken as the time of event occurrence. For infants who did not develop ROP, the date of the last normal fundus examination or the date of complete retinal vascularization was used as the censoring time. The secondary outcomes were the occurrence of IVH (any grade), hsPDA, BPD (any grade), LOS, and NEC. Severe ROP was defined as ROP stage ≥3 in either eye according to the ICROP criteria and was analyzed as a prespecified exploratory ophthalmic outcome.

### 2.8 Statistical analysis

Data organization and statistical analyses were performed using SPSS 25.0 (IBM Corp., Armonk, NY, USA) and R 4.4.2 (R Foundation for Statistical Computing, Vienna, Austria). (1) Handling of missing data: Variables with a missing rate > 20% were excluded. For continuous and categorical variables with a missing rate ≤ 20%, multiple imputation was performed using the mice package (Multiple Imputation by Chained Equations) in R software, with 5 imputations, and the pooled estimates across the 5 imputations were used for subsequent analyses. (2) Comparison of baseline characteristics: Continuous variables with a normal distribution were expressed as mean ± SD (x¯ ± s); between-group comparisons were performed using the independent-samples t test when variances were equal, or the corrected (Welch's) t test when variances were unequal. Continuous variables with a non-normal distribution were expressed as median (Q₁, Q₃) and compared using the Mann–Whitney U test. Categorical variables were expressed as counts (percentages) and compared using the *χ*^2^ test or Fisher's exact test (when the expected frequency was < 5). Correlations between continuous variables were assessed using Spearman correlation analysis. Multicollinearity in regression models was evaluated using the variance inflation factor (VIF), with VIF < 5 considered to indicate no significant collinearity.

Multivariable Cox regression analysis: With ROP occurrence as the outcome event, the time from birth to ROP diagnosis, death, transfer to another hospital, or the last follow-up as the follow-up time, and hyperglycemia exposure as the primary exposure of interest, four sequentially adjusted Cox proportional hazards regression models were constructed (results shown in Table 2). The Crude Model assessed the association between hyperglycemia exposure and ROP without adjustment for any covariates. Model 2 further adjusted for perinatal characteristics based on the Crude Model, including infant sex, GA, birth weight, SGA, maternal age, maternal pre-delivery BMI, mode of delivery, multiple birth (≥2 fetuses), antenatal full-course dexamethasone use, conception via assisted reproductive technology, duration of PROM, and 5-minute Apgar score. Model 3 additionally adjusted for maternal comorbidities based on Model 2, including maternal diabetes mellitus (including gestational diabetes mellitus and pregestational diabetes), maternal hypertension (including gestational hypertension and pregestational hypertension), and maternal thyroid disease. Model 4 additionally adjusted for neonatal complications based on Model 3, including IVH, hsPDA, BPD, LOS, and NEC. The results of each model are presented as hazard ratios (HRs) with 95% confidence intervals (CIs). The proportional hazards (PH) assumption was assessed using covariate-by-time interaction tests; if the PH assumption was violated, an extended Cox model with time-dependent covariates was used. Secondary outcomes were analyzed using logistic regression, and the results are expressed as odds ratios (ORs) with 95% CIs. A two-sided *P* < 0.05 was considered statistically significant. To evaluate the robustness of the association to respiratory support and glucose infusion rate (GIR), three additional models were constructed based on Model 4. The main analysis (Model 5) additionally adjusted for the duration of invasive mechanical ventilation, the duration of non-invasive ventilation, and the mean GIR; these three covariates showed no substantial collinearity (pairwise Spearman *ρ* ≤ 0.395; Supplementary Table S1). Two sensitivity analyses were performed to test whether the results were dependent on the specification of ventilation variables: Model 5b replaced the ventilation-type variables with the total duration of oxygen therapy and peak inspired oxygen fraction (FiO₂), together with GIR; Model 5c retained the duration of non-invasive ventilation and peak FiO₂, together with GIR, to verify that the association did not rely on the inclusion of invasive ventilation (Supplementary Table S2). For sparse-event secondary outcomes (severe ROP, *n* = 13), Firth penalized logistic regression (logistf, R 4.4.2) was applied to mitigate small-sample bias, with the crude OR also reported using Haldane's continuity correction. Because Model 5 included 24 covariates with 89 events (events-per-variable ratio ≈ 3.7), a Firth penalized Cox model (coxphf, R 4.4.2) was additionally fitted to address sparse-data bias. Covariates were selected *a priori* based on published evidence and clinical plausibility rather than data-driven selection. For the dose-response analysis, peak blood glucose was stratified into four categories (≤7, >7–8.33, >8.33–10, and >10 mmol/L), and Cox models were fitted under Model 3 and Model 4 adjustment. The linear trend was tested by entering the glucose category as a continuous covariate (one-degree-of-freedom Wald test), and deviation from linearity was assessed using a likelihood-ratio test comparing the categorical versus continuous specifications. The dose-response analysis was additionally performed under Model 5 adjustment as a sensitivity analysis (Supplementary Table S4); because GIR may partly lie on the causal pathway between glucose infusion and hyperglycemia, the Model 5-based estimates were considered conservative.

## 3 Results

### 3.1 General characteristics

During the study period, a total of 333 preterm infants with gestational age <32 weeks were screened. Eight cases with incomplete clinical data, 4 cases with genetic metabolic diseases, 2 cases with abnormal brain development, 2 cases with hereditary eye diseases, 4 cases who died before ROP screening could be completed, and 5 cases lost to follow-up due to transfer were excluded, resulting in a final inclusion of 308 cases(Figure 1). Among them, there were 172 males (55.8%) and 136 females (44.2%); the median birth weight was 1,365 (1141.3–1617.5) g and the median gestational age was 30.49 (29.14–31.53) weeks; a total of 89 cases developed ROP, with an overall incidence rate of 28.9%.

**Figure 1:**
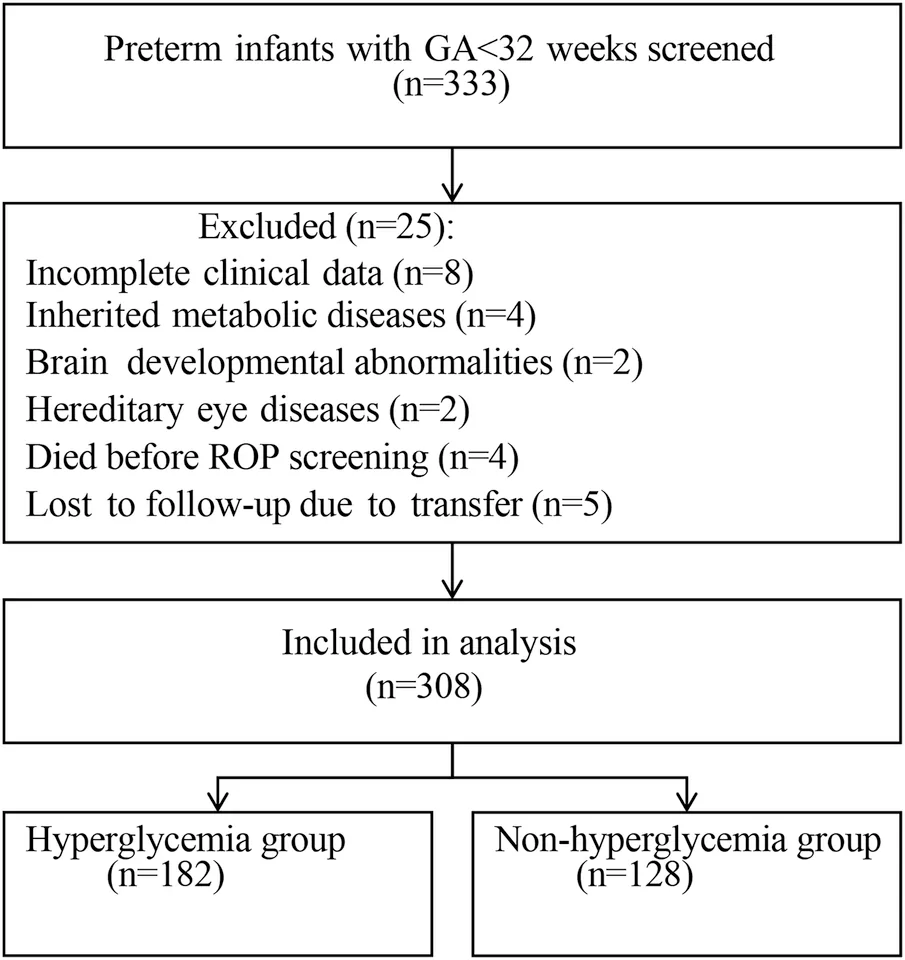
Flow diagram of study design.

### 3.2 Baseline characteristic analysis

A total of 308 preterm infants were included in this study, including 182 cases in the hyperglycemia group and 126 cases in the non-hyperglycemia group. The comparison of baseline characteristics between the two groups is shown in Table 1. The median birth weight [M (P25, P75)] in the hyperglycemia group was lower than that in the non-hyperglycemia group [1260 (1080, 1492.5) g vs. 1470 (1280, 1700) g, Z = −4.846, *P* < 0.001], and the median gestational age was also lower than that in the non-hyperglycemia group [30.14 (29.00, 31.14) weeks vs. 31.14 (29.97, 31.70) weeks, Z = −4.242, *P* < 0.001]. The pre-delivery BMI of mothers in the hyperglycemia group was higher than that in the non-hyperglycemia group [26.65 (24.40, 29.68) vs. 25.70 (23.48, 26.00), Z = −3.544, *P* < 0.001]. The proportion of multiple pregnancies (≥2 fetuses) (25.3% vs. 9.5%, *χ*^2^ = 12.085, *P* = 0.001) and the proportion of assisted reproduction (9.9% vs. 2.4%, *χ*^2^ = 6.608, *P* = 0.010) were both higher in the hyperglycemia group than in the non-hyperglycemia group. Regarding respiratory support, infants in the hyperglycemia group received longer invasive mechanical ventilation [3.00 (0.00, 5.00) vs. 0.00 (0.00, 1.67) d, *P* < 0.001], longer non-invasive ventilation [14.00 (7.00, 26.56) vs. 9.00 (4.13, 21.06) d, *P* = 0.002], longer total oxygen therapy [29.50 (15.00, 45.00) vs. 15.50 (7.75, 32.00) d, *P* < 0.001], and higher peak FiO₂ [40.00 (30.00, 45.00) vs. 30.00 (30.00, 40.00)%, *P* < 0.001] than those in the non-hyperglycemia group, whereas the mean glucose infusion rate was comparable between the two groups (*P* = 0.156). Regarding neonatal complications, the incidence rates of IVH (37.9% vs. 19.0%, *χ*^2^ = 12.571, *P* < 0.001), hs-PDA (13.7% vs. 5.6%, *χ*^2^ = 5.352, *P* = 0.021), BPD (48.9% vs. 26.2%, *χ*^2^ = 16.054, *P* < 0.001), and ROP (39.6% vs. 13.5%, *χ*^2^ = 24.626, *P* < 0.001) in the hyperglycemia group were all significantly higher than those in the non-hyperglycemia group. There were no statistically significant differences between the two groups in terms of gender, SGA, 5-minute Apgar score, maternal age, duration of premature rupture of membranes, cesarean section, maternal diabetes, maternal hypertension, maternal hypothyroidism, adequate antenatal use of glucocorticoids, glucose infusion rate, NEC, and late-onset sepsis (*P* > 0.05).

**Table 1:** Baseline characteristics and neonatal outcomes of the hyperglycemia and non-hyperglycemia groups (the table is presented in two panels: baseline characteristics and neonatal outcomes).

Variable	Whole population *N* = 308	Hyperglycemia group (*n* = 182)	Non-hyperglycemia group (*n* = 126)	*P* value
Baseline characteristics				
Male	55.8%	53.8%	58.7%	0.396 (Chi-Square test)
Female	44.2%	46.2%	41.3%	
Birth weight (g)	1,365 [1141.3, 1617.5]	1,260 [1080, 1492.5]	1,470 [1280, 1700]	**< 0.001** (Mann–Whitney U test)
Gestational age (weeks)	30.49 [29.14, 31.53]	30.14 [29, 31.14]	31.14 [29.97, 31.70]	**< 0.001** (Mann–Whitney U test)
5-minute Apgar score	9.00 [9.00, 9.00]	9.00 [9.00, 9.00]	9.00 [9.00, 9.00]	0.111 (Mann–Whitney U test)
Maternal age (years)	30.00 [26.00, 33.00]	30.00 [27, 33]	30.00 [25, 33]	0.094 (Mann–Whitney U test)
Maternal body mass index (kg/m^2^)	26.00 [24.13, 28.08]	26.65 [24.40, 29.68]	25.70 [23.48, 26.00]	**< 0.001** (Mann–Whitney U test)
Duration of PROM (hours)	0.00 [0.00, 3.00]	0.00 [0.00, 1.00]	0.00 [0.00, 7.00]	0.214 (Mann–Whitney U test)
Glucose infusion rate (24-h mean before glucose-lowering management, mg/kg/min)	6.29 [5.66, 6.74]	6.29 [5.70, 6.62]	6.31 [5.62, 6.86]	0.156 (Mann–Whitney U test)
Duration of invasive mechanical ventilation (d)	1.00 [0.00, 4.00]	3.00 [0.00, 5.00]	0.00 [0.00, 1.67]	**< 0.001** (Mann–Whitney U test)
Duration of non-invasive mechanical ventilation (d)	12.00 [5.47, 25.00]	14.00 [7.00, 26.56]	9.00 [4.13, 21.06]	**0.002** (Mann–Whitney U test)
Total duration of oxygen therapy (d)	23.00 [10.00, 38.00]	29.50 [15.00, 45.00]	15.50 [7.75, 32.00]	**< 0.001** (Mann–Whitney U test)
Peak FiO₂ (%)	35.00 [30.00, 45.00]	40.00 [30.00, 45.00]	30.00 [30.00, 40.00]	**< 0.001** (Mann–Whitney U test)
SGA	6.5%	8.2%	4.0%	0.135 *(Chi-Square test)*
Cesarean section	74.4%	77.5%	69.8%	0.132 (Chi-Square test)
Multiple birth (≥2)	18.8%	25.3%	9.5%	**0.001** (Chi-Square test)
Maternal diabetes	8.4%	8.3%	8.7%	0.888 (Chi-Square test)
Maternal hypertension	27.9%	31.9%	22.2%	0.064 (Chi-Square test)
Maternal hypothyroidism	5.8%	6.0%	5.6%	0.857 (Chi-Square test)
Assisted reproduction	6.8%	9.9%	2.4%	**0.010** (Chi-Square test)
Full-course antenatal corticosteroids	42.2%	45.1%	38.1%	0.224 (Chi-Square test)
Neonatal outcomes				
Intraventricular hemorrhage	30.2%	37.9%	19.0%	**< 0.001** (Chi-Square test)
Hemodynamically significant PDA	10.4%	13.7%	5.6%	**0.021** (Chi-Square test)
Necrotizing enterocolitis	11.0%	11.5%	10.3%	0.737 (Chi-Square test)
Bronchopulmonary dysplasia	39.6%	48.9%	26.2%	**< 0.001** (Chi-Square test)
Late-onset sepsis	4.9%	4.4%	5.5%	0.879 (Chi-Square test)
Retinopathy of prematurity	28.9%	39.6%	13.5%	**< 0.001** (Chi-Square test)
Severe ROP (stage ≥3)	4.2%	5.5%	2.4%	0.181 (Chi-Square test)

The bold values represent statistically significant results (*p* < 0.05).

### 3.3 Cox regression analysis

The time-dependent covariate test did not find obvious violations of the proportional hazards assumption (all *P* > 0.05). On this basis, multivariate Cox regression analysis was performed (Table 2). Without adjustment, the cumulative incidence of ROP in the hyperglycemia group was significantly higher than that in the non-hyperglycemia group (HR = 3.08, 95%CI: 1.81–5.22, *P* < 0.001); after adjusting for perinatal characteristics such as gender, gestational age, birth weight, SGA, maternal age, maternal BMI, mode of delivery, multiple birth (≥2 fetuses), adequate antenatal use of dexamethasone, assisted reproduction, duration of premature rupture of membranes, and 5-minute Apgar score (Model 2), hyperglycemia remained significantly associated with ROP (HR = 2.58, 95%CI: 1.49–4.46, *P* = 0.001); after further adjusting for maternal diabetes, hypertension, and thyroid diseases (Model 3), hyperglycemia remained an independent risk factor for ROP (HR = 2.57, 95%CI: 1.49–4.46, *P* = 0.001), with no obvious attenuation in the effect size compared to Model 2. Based on Model 3, after additionally including neonatal complications such as IVH, hsPDA, BPD, sepsis, and NEC, the association between hyperglycemia and ROP did not undergo substantial changes (HR = 2.54, 95%CI: 1.45–4.44), suggesting robust conclusions. To further address confounding by respiratory support and nutritional management (Supplementary Table S2), three additional models were constructed: after additional adjustment for the duration of invasive and non-invasive ventilation and the mean GIR (Model 5), hyperglycemia remained independently associated with ROP (HR = 2.46, 95%CI: 1.40–4.31, *P* = 0.002); similar results were obtained when the ventilation variables were replaced by the total duration of oxygen therapy and peak FiO₂ (Model 5b: HR = 2.23, 95%CI: 1.27–3.92, *P* = 0.003), and when invasive ventilation was omitted (Model 5c: HR = 2.26, 95%CI: 1.28–3.98, *P* = 0.005). Given the events-per-variable ratio (89 events/24 covariates ≈ 3.7), a Firth penalized Cox model was additionally fitted for Model 5 and yielded consistent results (HR = 2.36, 95%CI: 1.36–4.11, *P* = 0.001), indicating that the association was not attributable to sparse-data bias. Regarding the secondary outcomes, hyperglycemia was significantly associated with IVH across all three models, and the effect size increased after adjustment (Model 1: OR = 2.60, 95% CI: 1.52–4.44, *P* < 0.001; Model 3: OR = 2.68, 95% CI: 1.45–4.96, *P* = 0.002). This association remained statistically significant after correction for multiple testing using the Benjamini-Hochberg method (FDR-adjusted *P* = 0.010), suggesting a negative confounding effect. The crude associations between hyperglycemia and BPD (Model 1: OR = 2.70, 95% CI: 1.64–4.41, *P* < 0.001; Model 3: OR = 1.89, 95% CI: 0.96–3.75, *P* = 0.067) and between hyperglycemia and hsPDA (Model 1: OR = 2.71, 95% CI: 1.13–6.47, *P* = 0.025; Model 3: OR = 1.70, 95% CI: 0.63–4.56, *P* = 0.295) were attenuated after adjustment and were not significant after FDR correction. Hyperglycemia was not significantly associated with LOS (Model 3: OR = 1.11, 95% CI: 0.33–3.70, *P* = 0.871) or NEC (Model 3: OR = 1.56, 95% CI: 0.64–3.77, *P* = 0.330).

**Table 2:** Cox proportional hazards regression analysis of the association between hyperglycemia and retinopathy of prematurity (ROP).

Variable	Model 1 HR/OR (95%CI)	*P* value	Model 2 HR/OR (95%CI)	*P* value	Model 3 HR/OR (95%CI)	*P* value	FDR adjust *P* value	Model 4 HR (95%CI)	*P* value
Any ROP	3.08 (1.81−5.22)	< 0.001	2.58 (1.49–4.46)	0.001	2.57 (1.49–4.46)	0.001	-	2.54 (1.45–4.44)	0.001
IVH (any grade)	2.60 (1.52–4.44)	< 0.001	2.62 (1.42–4.83)	0.002	2.68 (1.45–4.96)	0.002	0.010	-	-
hs-PDA	2.71 (1.13–6.47)	0.025	1.71 (0.65–4.55)	0.280	1.70 (0.63–4.56)	0.295	0.413	-	-
BPD	2.70 (1.65–4.41)	< 0.001	1.86 (0.94–3.66)	0.073	1.89 (0.96–3.75)	0.067	0.168	-	-
LOS	0.92 (0.31–2.72)	0.879	1.10 (0.33–3.63)	0.880	1.11 (0.33–3.70)	0.871	0.871	-	-
NEC	1.13 (0.55–2.36)	0.737	1.55 (0.64–3.72)	0.330	1.56 (0.64–3.77)	0.330	0.413	-	-

Model 1: Unadjusted. Model 2: Model 1 plus adjustment for gender, gestational age, birth weight, small for gestational age (SGA), multiple birth (≥2), maternal age, maternal body mass index (BMI), cesarean section, full-course antenatal corticosteroids, assisted reproduction, duration of PROM, and 5-minute Apgar score. Model 3: Model 2 plus maternal diabetes mellitus, maternal hypertension, and maternal hypothyroidism. Model 4: Model 3 plus IVH, hsPDA, BPD, sepsis (late-onset sepsis), and NEC. Model 4 was estimated for the primary outcome (ROP) only, because the neonatal complications corresponding to the secondary outcomes are included as covariates in Model 4 and an outcome cannot be adjusted for itself. The primary outcome was excluded from false discovery rate (FDR) correction; *P* values of the five secondary outcomes were adjusted using the Benjamini–Hochberg method. The proportional hazards assumption was tested via covariate-by-time interaction terms and was satisfied for all covariates (all *P* > 0.05). HR, hazard ratio; OR, odds ratio; FDR, false discovery rate.

Severe ROP (stage ≥3) occurred in 10/182 (5.5%) infants in the hyperglycemia group and 3/126 (2.4%) in the non-hyperglycemia group. The crude association was not statistically significant (OR = 2.15, 95% CI: 0.58–7.97, *P* = 0.253, Haldane-corrected), and the estimate was further attenuated after Firth penalized adjustment for gestational age, birth weight, and sex (OR = 1.45, 95% CI: 0.36–7.31, *P* = 0.612). Owing to the small number of events (*n* = 13), these exploratory analyses have limited statistical power and should be interpreted with caution (Supplementary Table S3).

### 3.4 Sensitivity analysis

To further explore the dose-response relationship between the severity of hyperglycemia and the risk of ROP, the exposure variable was re-stratified according to peak blood glucose levels (Table 3). Using blood glucose ≤7 mmol/L (126 mg/dL) as the reference group, Cox regression analysis was performed based on Model 3 (adjusting for perinatal characteristics and maternal comorbidities). The results showed that as blood glucose levels increased, the risk of ROP showed a significant increasing trend: the risk of ROP in the group with blood glucose >7 mmol/L (126 mg/dL) and ≤8.33 mmol/L (150 mg/dL) (*n* = 42) did not show a significant increase (HR = 1.11, 95%CI: 0.45–2.76, *P* = 0.818); the risk of ROP in the group with blood glucose >8.33 mmol/L (150 mg/dL) and ≤10 mmol/L (180 mg/dL) (*n* = 60) increased significantly (HR = 2.72, 95%CI: 1.40–5.26, *P* = 0.003); the risk of ROP in the group with blood glucose >10 mmol/L (180 mg/dL) (*n* = 80) further increased (HR = 3.64, 95%CI: 1.96–6.78, *P* < 0.001), suggesting a dose-response relationship between hyperglycemia and ROP, where the higher the blood glucose level, the greater the risk of ROP. The trend test showed that the risk of ROP exhibited a significant linear increasing trend with the elevation of blood glucose grades (*P* for trend <0.001, with the risk of ROP increasing by an average of 79% for each grade increase in blood glucose), suggesting a clear dose-response relationship between hyperglycemia and ROP, where the higher the blood glucose level, the greater the risk of ROP. This dose-response pattern was robust to further adjustment for neonatal complications: under Model 4, the adjusted HRs were 1.55 (95%CI: 0.69–3.47), 2.66 (95%CI: 1.35–5.24), and 3.49 (95%CI: 1.88–6.47) across the three glucose categories, with a per-grade HR of 1.52 (95%CI: 1.25–1.85, *P* = 2.9 × 10⁻⁵). The trend did not deviate from linearity (*P* for non-linearity=0.87). In a further sensitivity analysis additionally adjusting for the duration of invasive and non-invasive ventilation and the mean glucose infusion rate (Model 5), the per-grade HR remained significant (HR = 1.50, 95%CI: 1.23–1.82, *P* = 5.6 × 10⁻⁵; Supplementary Table S4); given that the glucose infusion rate may partly lie on the causal pathway to hyperglycemia, this estimate should be regarded as conservative.

**Table 3:** Dose-response analysis of the association between hyperglycemia severity and the risk of retinopathy of prematurity.

Peak blood glucose	Model 3 HR (95%CI)	*P* value	Model 4 HR (95%CI)	*P* value
Blood glucose ≤7 mmol/L (126 mg/dL) (*n* = 126)	1.00 (ref)		1.00 (ref)	
Blood glucose >7 mmol/L (126 mg/dL) & ≤ 8.33 mmol/L (150 mg/dL) (*n* = 42)	1.11 (0.45–2.76)	0.818	1.55 (0.69–3.47)	0.287
Blood glucose >8.33 mmol/L (150 mg/dL) & ≤ 10 mmol/L (180 mg/dL) (*n* = 60)	2.72 (1.40–5.26)	0.003	2.66 (1.35–5.24)	0.005
Blood glucose >10 mmol/L (180 mg/dL) (*n* = 80)	3.64 (1.96–6.78)	< 0.001	3.49 (1.88–6.47)	< 0.001
*P* for linear trend (per grade)	1.79 (1.48–2.18)	< 0.001	1.52 (1.25–1.85)	< 0.001
*P* for non-linearity	0.93		0.87	

Model 3: adjusted for perinatal characteristics and maternal comorbidities (identical to Model 3 in Table 2). Model 4: further adjusted for neonatal complications including IVH, hsPDA, BPD, late-onset sepsis, and NEC. Model 3 estimates were obtained with SPSS 25.0. Model 4 estimates, the linear trend test (blood glucose category entered as a continuous covariate, one-degree-of-freedom Wald test), and the test for non-linearity (likelihood-ratio test comparing the categorical versus continuous specification) were obtained with R 4.4.2 (survival package), using Breslow's method for tied events, which is identical to the SPSS algorithm. Because of the multiple-imputation workflow, the two implementations yielded slightly different point estimates (e.g., per-grade HR 1.79 vs. 1.52) without affecting the direction, magnitude, or significance of any association. The dose-response analysis was additionally verified under Model 5 adjustment (Supplementary Table S4).

## 4 Discussion

Retinopathy of prematurity (ROP) is a leading cause of preventable childhood blindness worldwide (3). It affects premature infants through aberrant retinal vascular development that can progress to retinal detachment and severe visual impairment (24). Despite advances in neonatal care, the global incidence of ROP continues to rise as survival rates of extremely preterm infants improve. This trend places a substantial burden on healthcare systems and families. Current screening relies on serial ophthalmoscopic examinations. Treatment options include laser photocoagulation and intravitreal anti-vascular endothelial growth factor (VEGF) injections. These interventions, however, are invasive and costly, and their long-term outcomes remain uncertain (25, 26). These limitations underscore an urgent need to identify modifiable risk factors for early prevention.

This multicenter retrospective cohort study of 308 preterm infants with gestational age <32 weeks investigated the independent association between neonatal hyperglycemia and ROP risk. Rigorous multivariable Cox regression models were employed to adjust for perinatal characteristics, maternal comorbidities, and neonatal complications. Our findings demonstrate that hyperglycemia is an independent risk factor for ROP (HR = 2.54, 95%CI: 1.45–4.44). This association persisted after additional adjustment for respiratory support variables and glucose infusion rate (HR = 2.46, 95%CI: 1.40–4.31) and in Firth penalized sensitivity analysis (HR = 2.36, 95%CI: 1.36–4.11), indicating that it was not explained by oxygen therapy, ventilatory support, or nutritional management. A significant dose-response relationship was observed, with ROP risk increasing by approximately 50%–80% for each incremental elevation in blood glucose level across model specifications (per-grade HR 1.50–1.79). This gradient was unchanged after further adjustment for neonatal complications, respiratory support, and glucose infusion rate, and showed no significant deviation from linearity (*P* for non-linearity ≥ 0.86). Furthermore, hyperglycemia was independently associated with intraventricular hemorrhage (IVH) (OR = 2.68, 95%CI: 1.45–4.96), suggesting a broader systemic vascular effect. These results provide robust evidence supporting the clinical importance of strict glycemic control in preterm infants and highlight the potential for blood glucose thresholds to guide ROP screening and prevention strategies.

This study presents several novel findings that advance the understanding of the relationship between neonatal hyperglycemia and ROP. While previous research has suggested a link between hyperglycemia and ROP, our study comprehensively quantify the independent, dose-response relationship between hyperglycemia severity and ROP risk using a rigorous multicenter cohort design and extensive covariate adjustment. Unlike earlier investigations that often suffered from insufficient confounding control, our stepwise adjustment for perinatal characteristics, maternal comorbidities, and neonatal complications demonstrated that the association remained robust, effectively isolating hyperglycemia as an independent risk factor. The key innovation lies in the sensitivity analysis, which revealed a clear gradient effect: compared to normoglycemic infants, those with blood glucose levels >8.33 mmol/L (150 mg/dL) had significantly increased ROP risk (HR = 2.72), and this risk more than tripled in the >10 mmol/L (180 mg/dL) group (HR = 3.64). This quantifiable threshold provides a clinically actionable target, moving beyond binary classification toward graded risk assessment. This dose-response relationship is biologically plausible. Hyperglycemia concentration-dependently promotes the accumulation of advanced glycation end-products (AGEs) and induces oxidative stress, which in turn upregulates VEGF and triggers abnormal retinal neovascularization-a core pathophysiological mechanism of ROP (27, 28). Our findings are compatible with a causal role of hyperglycemia but cannot establish causation (29, 30), offering a more precise framework for risk stratification than previously available.

The clinical implications are substantial and directly actionable for NICUs. The identification of a clear glycemic threshold above which ROP risk escalates dramatically provides a concrete, evidence-based target for intervention. Implementing strict glycemic control protocols to maintain blood glucose below this threshold could be an effective strategy for primary prevention of ROP in at-risk preterm infants, particularly those born before 32 weeks of gestation (19, 20). Furthermore, the independent association between hyperglycemia and IVH underscores the multi-systemic deleterious effects of hyperglycemia, likely mediated through common pathways of endothelial dysfunction and hemodynamic instability (31, 32). This dual risk profile highlights that effective glucose management is not merely an adjunctive measure, but a central component of a comprehensive strategy to mitigate the most severe neurological and ophthalmological complications of prematurity (10). Our findings support a paradigm shift in NICU care-from passive monitoring of hyperglycemia to proactive, protocol-driven management. We suggest that infants with peak blood glucose levels exceeding 8.33 mmol/L (150 mg/dL) be prioritized for more frequent ROP screening and closer glycemic surveillance. Because insulin treatment was not systematically documented in this retrospective cohort, and insulin therapy in preterm infants carries a well-recognized risk of iatrogenic hypoglycemia (33, 34), our findings do not justify a specific insulin initiation threshold; this question requires prospective evaluation with continuous glucose monitoring. This approach has the potential to significantly reduce the burden of ROP-related blindness and improve long-term neurodevelopmental outcomes (35).

The robustness of our conclusions is underpinned by a meticulous and transparent analytical strategy. The stepwise Cox regression models systematically accounted for confounding, starting with perinatal factors (Model 2), then maternal comorbidities (Model 3), and finally neonatal complications (Model 4). The hazard ratio for hyperglycemia remained remarkably stable (from HR = 2.58 to HR = 2.54) across these models rather than being attenuated, and remained essentially unchanged after further adjustment for respiratory support and glucose infusion rate (HR = 2.46) and in Firth penalized analysis (HR = 2.36). This strongly suggests that hyperglycemia exerts an independent effect on ROP (36), not merely serving as a proxy for other known risk factors such as low birth weight or low gestational age (17, 18). This approach is methodologically sound (37), as neonatal complications such as sepsis and bronchopulmonary dysplasia (BPD) could act as both confounders and mediators on the causal pathway (38). By including them in the final model, we provide the most conservative estimate of the effect, confirming its independence (39). Furthermore, rigorous testing of the proportional hazards assumption-time-dependent covariate tests-validated the use of the Cox model and ensured that our estimates were not biased by time-dependent effects. This level of analytical rigor is essential for establishing causality in observational research (40). Our findings provide solid observational evidence derived from retrospective analysis, which cannot alone warrant revisions to formal clinical guidelines. Nevertheless, the results support considering hyperglycemia as an important modifiable risk factor for incorporation into ROP predictive models and clinical management regimens pending further prospective validation.

Several limitations warrant consideration. First, the retrospective multicenter design inherently carries risks of selection and information bias. Notably, intermittent hyperglycemic episodes may have been missed due to the fixed frequency of blood glucose monitoring (three times daily), potentially underestimating the true exposure burden. Second, the inclusion criterion of a hospitalization duration ≥4 weeks may have introduced survivor bias: 4 screened infants died and 5 were transferred before ROP screening could be completed, and these infants are not represented in the cohort. Infants with the greatest immaturity and critical illness are at the highest risk of hyperglycemia. Since these infants account for a substantial proportion of early deaths and patient transfers, the observed association may underestimate the true risk of retinopathy of prematurity (ROP) attributable to hyperglycemia. Nevertheless, the direction of this bias cannot be ascertained using retrospective data. Third, insulin therapy and detailed nutritional management were not systematically documented, precluding assessment of treatment-related confounding. Fourth, with 89 ROP events and up to 24 covariates in the extended models, the events-per-variable ratio (≈3.7) is below the conventional benchmark of 10. Although multicollinearity was formally assessed (all VIF < 5) and Firth penalized sensitivity analyses yielded consistent results, some degree of model overfitting cannot be excluded. Fifth, the absence of continuous glucose monitoring (CGM) data precluded an assessment of glycemic variability, a metric increasingly recognized for its impact on vascular outcomes (41, 42). Sixth, although the overall sample size satisfied pre-specified power calculations, some subgroups within the dose-response analysis such as mild hyperglycemia had relatively small sample sizes, which may compromise statistical precision. Furthermore, the limited count of severe ROP events (*n* = 13) prevented any adequately-powered analysis focused on severe ROP outcomes. Finally, as the cohort was drawn exclusively from Ningxia, external validation in diverse geographic populations is necessary before generalizing these thresholds globally. The lack of complementary wet-lab experiments also restricts our ability to definitively establish the molecular mechanisms underlying the observed clinical associations.

In conclusion, this multicenter cohort study provides robust evidence that neonatal hyperglycemia is an independent risk factor for ROP in infants born before 32 weeks of gestation. We delineated a significant dose-response relationship and identified 8.33 mmol/L (150 mg/dL) as a critical threshold for clinical vigilance. The independent association between hyperglycemia and IVH further underscores its role in multi-organ vascular injury among preterm neonates. These findings advocate for stringent glycemic management protocols and prioritized ROP screening for hyperglycemic infants. Future prospective studies integrating CGM technology and mechanistic explorations are imperative to refine metabolic strategies and ultimately mitigate the burden of vision-threatening complications in this vulnerable population (35).

## Data Availability

The raw data supporting the conclusions of this article will be made available by the authors, without undue reservation.

## Supplementary material

Supplementary Table S1 Spearman correlation coefficients among the continuous variables included in the multivariable models.

Supplementary Table S2 Extended Cox proportional hazards models additionally adjusted for respiratory support and glucose infusion rate (Models 5, 5b and 5c), together with the Firth penalized Cox model for the primary outcome.

Supplementary Table S3 Association between neonatal hyperglycemia and severe retinopathy of prematurity (stage ≥3): crude odds ratio with Haldane's continuity correction and Firth penalized logistic regression.

Supplementary Table S4 Dose-response analysis of peak blood glucose level and retinopathy of prematurity under Models 3, 4 and 5 adjustment, including the linear trend and non-linearity tests.
