# Supplementary material for: Neonatal hyperglycemia as an independent risk factor for retinopathy of prematurity: a multicenter retrospective cohort study with dose-response analysis
Source: Front Pediatr. 2026 Oct 23;14:1975596. doi: 10.3389/fped.2026.1975596 (PMC13739780; doi:10.3389/fped.2026.1975596)
Supplement: Supplementary file 1 [file Table1.docx]

**Supplementary Table S1. Spearman correlation coefficients among the continuous variables included in the multivariable models**

| **Variable** | **Duration of invasive mechanical ventilation (d)** | **Duration of non-invasive ventilation (d)** | **Total oxygen therapy (d)** | **Peak FiO₂** | **GIR (mg/kg/min)** |
| --- | --- | --- | --- | --- | --- |
| Duration of invasive mechanical ventilation (d) | 1.00 | 0.395 | 0.481 | 0.862 | -0.10 |
| Duration of non-invasive ventilation (d) | 0.395 | 1.00 | 0.777 | 0.397 | -0.05 |
| Total oxygen therapy (d) | 0.481 | 0.777 | 1.00 | 0.418 | -0.07 |
| Peak FiO₂ | 0.862 | 0.397 | 0.418 | 1.00 | -0.08 |
| GIR (mg/kg/min) | -0.10 | -0.05 | -0.07 | -0.08 | 1.00 |

GIR showed no substantial correlation with any respiratory support variable (all |ρ| ≤ 0.10); among the respiratory variables, invasive mechanical ventilation and peak FiO₂ were strongly correlated (ρ = 0.862), as were non-invasive ventilation and total oxygen therapy (ρ = 0.777). Therefore, invasive ventilation, non-invasive ventilation, and GIR were jointly entered into Model 5 (pairwise ρ ≤ 0.395 among the three), while peak FiO₂ and total oxygen therapy were used in alternative sensitivity specifications (Models 5b and 5c).
