# Supplementary material for: Neonatal hyperglycemia as an independent risk factor for retinopathy of prematurity: a multicenter retrospective cohort study with dose-response analysis
Source: Front Pediatr. 2026 Oct 23;14:1975596. doi: 10.3389/fped.2026.1975596 (PMC13739780; doi:10.3389/fped.2026.1975596)
Supplement: Supplementary file 2 [file Table2.docx]

**Supplementary Table S2. Extended Cox proportional hazards models additionally adjusted for respiratory support and glucose infusion rate (Models 5, 5b and 5c), together with the Firth penalized Cox model for the primary outcome**

| **Variable** | **Model 1 HR (95%CI)** | **P** | **Model 2 HR (95%CI)** | **P** | **Model 3 HR (95%CI)** | **P** | **Model 4 HR (95%CI)** | **P** | **Model 5 HR (95%CI)** | **P** | **Model 5b HR (95%CI)** | **P** | **Model 5c HR (95%CI)** | **P** | **Firth-M5 HR (95%CI)** | **P** |
| --- | --- | --- | --- | --- | --- | --- | --- | --- | --- | --- | --- | --- | --- | --- | --- | --- |
| Any ROP | 3.08 (1.81–5.22) | <0.001 | 2.58 (1.49–4.46) | 0.001 | 2.57 (1.49–4.46) | 0.001 | 2.54 (1.45–4.44) | 0.001 | 2.46 (1.40–4.31) | 0.002 | 2.23 (1.27–3.92) | 0.003 | 2.26 (1.28–3.98) | 0.005 | 2.36 (1.36–4.11) | 0.001 |

Model 1: Unadjusted. Model 2: Model 1 plus adjustment for gender, gestational age, birth weight, small for gestational age (SGA), multiple birth (≥2), maternal age, maternal body mass index (BMI), cesarean section, full-course antenatal corticosteroids, assisted reproduction, duration of PROM, and 5-minute Apgar score. Model 3: Model 2 plus maternal diabetes mellitus, maternal hypertension, and maternal hypothyroidism. Model 4: Model 3 plus IVH, hsPDA, BPD, sepsis (late-onset sepsis), and NEC. Model 5 (main): Model 4 plus duration of invasive mechanical ventilation, duration of non-invasive ventilation, and glucose infusion rate (pairwise Spearman ρ ≤ 0.395, indicating no significant collinearity). Model 5b (sensitivity): Model 4 plus total duration of oxygen therapy, peak FiO₂, and glucose infusion rate, replacing ventilation-mode variables with overall oxygen exposure. Model 5c (sensitivity): Model 4 plus duration of non-invasive ventilation, peak FiO₂, and glucose infusion rate, to confirm that the association was not dependent on invasive ventilation. Firth-M5: Firth penalized Cox regression (coxphf, R 4.4.2) using the Model 5 specification, to address sparse-data bias (89 events / 24 covariates).

HR = hazard ratio. Hyperglycemia remained independently associated with ROP in all extended models and in the Firth penalized sensitivity analysis.
