# Supplementary material for: Neonatal hyperglycemia as an independent risk factor for retinopathy of prematurity: a multicenter retrospective cohort study with dose-response analysis
Source: Front Pediatr. 2026 Oct 23;14:1975596. doi: 10.3389/fped.2026.1975596 (PMC13739780; doi:10.3389/fped.2026.1975596)
Supplement: Supplementary file 3 [file Table3.docx]

**Supplementary Table S3. Association between neonatal hyperglycemia and severe retinopathy of prematurity (stage ≥3): crude odds ratio with Haldane's continuity correction and Firth penalized logistic regression**

| **Outcome** | **Hyperglycemia n/N (%)** | **Normoglycemia n/N (%)** | **Crude OR* (95% CI), P value** | **Adjusted OR† (95% CI), P value** |
| --- | --- | --- | --- | --- |
| Severe ROP (stage ≥3) | 10/182 (5.5%) | 3/126 (2.4%) | 2.15 (0.58–7.97), P=0.253 | 1.45 (0.36–7.31), P=0.612 |

* Crude odds ratio with Haldane's continuity correction (sparse events). † Firth penalized logistic regression, adjusted for gestational age, birth weight, and sex. Given the small number of events (n=13), these analyses were considered exploratory and should be interpreted with caution.

**Supplementary Table S4.Dose-response analysis of peak blood glucose level and retinopathy of prematurity under Models 3, 4 and 5 adjustment, including the linear trend and non-linearity tests**

| **Peak blood glucose** | **Model 3 HR (95%CI), P** | **Model 4 HR (95%CI), P** | **Model 5 HR (95%CI), P** |
| --- | --- | --- | --- |
| ≤7 mmol/L (126 mg/dL) (n=126) | 1.00 (ref) | 1.00 (ref) | 1.00 (ref) |
| >7–8.33 mmol/L (126–150 mg/dL) (n=42) | 1.56 (0.71–3.45), P=0.272 | 1.55 (0.69–3.47), P=0.287 | 1.54 (0.68–3.46), P=0.299 |
| >8.33–10 mmol/L (150–180 mg/dL) (n=60) | 2.56 (1.33–4.92), P=0.005 | 2.66 (1.35–5.24), P=0.005 | 2.60 (1.31–5.13), P=0.006 |
| >10 mmol/L (180 mg/dL) (n=80) | 3.53 (1.93–6.46), P<0.001 | 3.49 (1.88–6.47), P<0.001 | 3.35 (1.80–6.21), P<0.001 |
| Per-grade HR (linear trend) | 1.52 (1.26–1.84), P=1.6×10⁻⁵ | 1.52 (1.25–1.85), P=2.9×10⁻⁵ | 1.50 (1.23–1.82), P=5.6×10⁻⁵ |
| P for non-linearity | 0.93 | 0.87 | 0.86 |

All estimates in this table were computed with R 4.4.2 (survival package, Breslow method for tied events), providing a within-software comparison across models. Model specifications are identical to Table 2 of the manuscript. The linear trend was tested by entering the blood glucose category as a continuous covariate (one-degree-of-freedom Wald test); P for non-linearity was obtained from a likelihood-ratio test comparing the categorical versus continuous specifications.

The dose-response gradient was virtually unchanged across Model 3, Model 4 (neonatal complications), and Model 5 (respiratory support and GIR) adjustment, and showed no significant deviation from linearity. Because the glucose infusion rate may partly lie on the causal pathway between glucose infusion and hyperglycemia, the Model 5-based estimates should be regarded as conservative.
